# Composition Optimization and Microstructure Design in MOFs-Derived Magnetic Carbon-Based Microwave Absorbers: A Review

**DOI:** 10.1007/s40820-021-00734-z

**Published:** 2021-10-11

**Authors:** Honghong Zhao, Fengyuan Wang, Liru Cui, Xianzhu Xu, Xijiang Han, Yunchen Du

**Affiliations:** MIIT Key Laboratory of Critical Materials Technology for New Energy Conversion and Storage, School of Chemistry and Chemical Engineering, Harbin Institute of Technology, Harbin, 150001 People’s Republic of China

**Keywords:** Magnetic carbon-based composites, Metal–organic frameworks, Composition optimization, Microstructure design, EM absorption enhancement

## Abstract

Magnetic carbon-based composites are the most attractive candidates for electromagnetic (EM) absorption because they can terminate the propagation of surplus EM waves in space by interacting with both electric and magnetic branches. Metal-organic frameworks (MOFs) have demonstrated their great potential as sacrificing precursors of magnetic metals/carbon composites, because they provide a good platform to achieve high dispersion of magnetic nanoparticles in carbon matrix. Nevertheless, the chemical composition and microstructure of these composites are always highly dependent on their precursors and cannot promise an optimal EM state favorable for EM absorption, which more or less discount the superiority of MOFs-derived strategy. It is hence of great importance to develop some accompanied methods that can regulate EM properties of MOFs-derived magnetic carbon-based composites effectively. This review comprehensively introduces recent advancements on EM absorption enhancement in MOFs-derived magnetic carbon-based composites and some available strategies therein. In addition, some challenges and prospects are also proposed to indicate the pending issues on performance breakthrough and mechanism exploration in the related field.

## Highlights

- This review introduces recent advances to optimize electromagnetic properties of metal-organic frameworks (MOFs)-derived magnetic carbon-based composites through rational microstructure design and composition optimization in detail.
- The challenges and outlooks in MOFs-derived magnetic carbon-based microwave absorbers are also proposed and analyzed, including low-frequency absorption, diversified MOFs precursors, structure-activity relationships, environmental tolerance.

## Graphical abstract

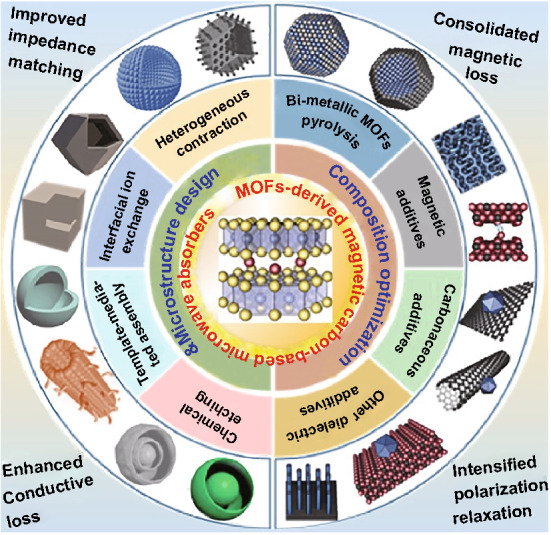

## Introduction

The worsening electromagnetic (EM) environment caused by EM radiation from the massive usages of emerging electronic apparatuses ranging from household appliances to wireless base stations and military radars poses serious threats to human health and national defense security and has aroused worldwide attention [1–3]. EM shielding and EM absorption have long been recognized as two typical strategies for mitigating or resisting adverse effects from those surplus EM waves, where the former realizes individual protection through strong reflection of incident EM waves and the latter is established on the conversion of EM energy [4, 5]. In view of their different mechanisms, EM absorption has gradually evolved into a dominant means for EM pollution precaution due to its desirable sustainability [6, 7]. The key point of EM absorption is to interrupt the transmission of EM waves by interacting with their magnetic field branch or electric field branch, and thus, some functional materials with good EM characteristics, i.e., magnetic and dielectric properties, are usually considered as promising microwave absorbers [8–10]. In an effort to achieve considerable absorption performance, the integration of magnetic and dielectric media, especially for composites with magnetic metal/alloy particles and carbon materials, becomes an overwhelming mode in the development of microwave absorbers [6, 11, 12]. On the one hand, magnetic metals/alloys have higher saturation magnetization than magnetic ferrites, and thus, they can produce distinguishable permeability and strong magnetic response in the frequency range of gigahertz [11, 13]. On the other hand, carbon materials have tunable dielectric property, good chemical stability, low density, and diversified morphology and microstructure, which render them as one of the most attractive components in composites for EM absorption [14, 15]. Conventional magnetic carbon-based composites are generally fabricated by decorating carbon materials with magnetic metal/alloy particles or high-temperature pyrolysis of polymers containing various magnetic precursors (e.g., metal salts and oxides) [16–18]. However, the products from these routes widely suffer from disordered microstructures, poor chemical homogeneity, random nanoparticle size, and dispersion, which may weaken the synergy between magnetic particles and carbon matrix to some extent [19]. Therefore, an effective strategy that can promise significant improvements on the shortcomings mentioned above is extremely desirable for high-performance magnetic carbon-based composites.

**Fig. 1:**
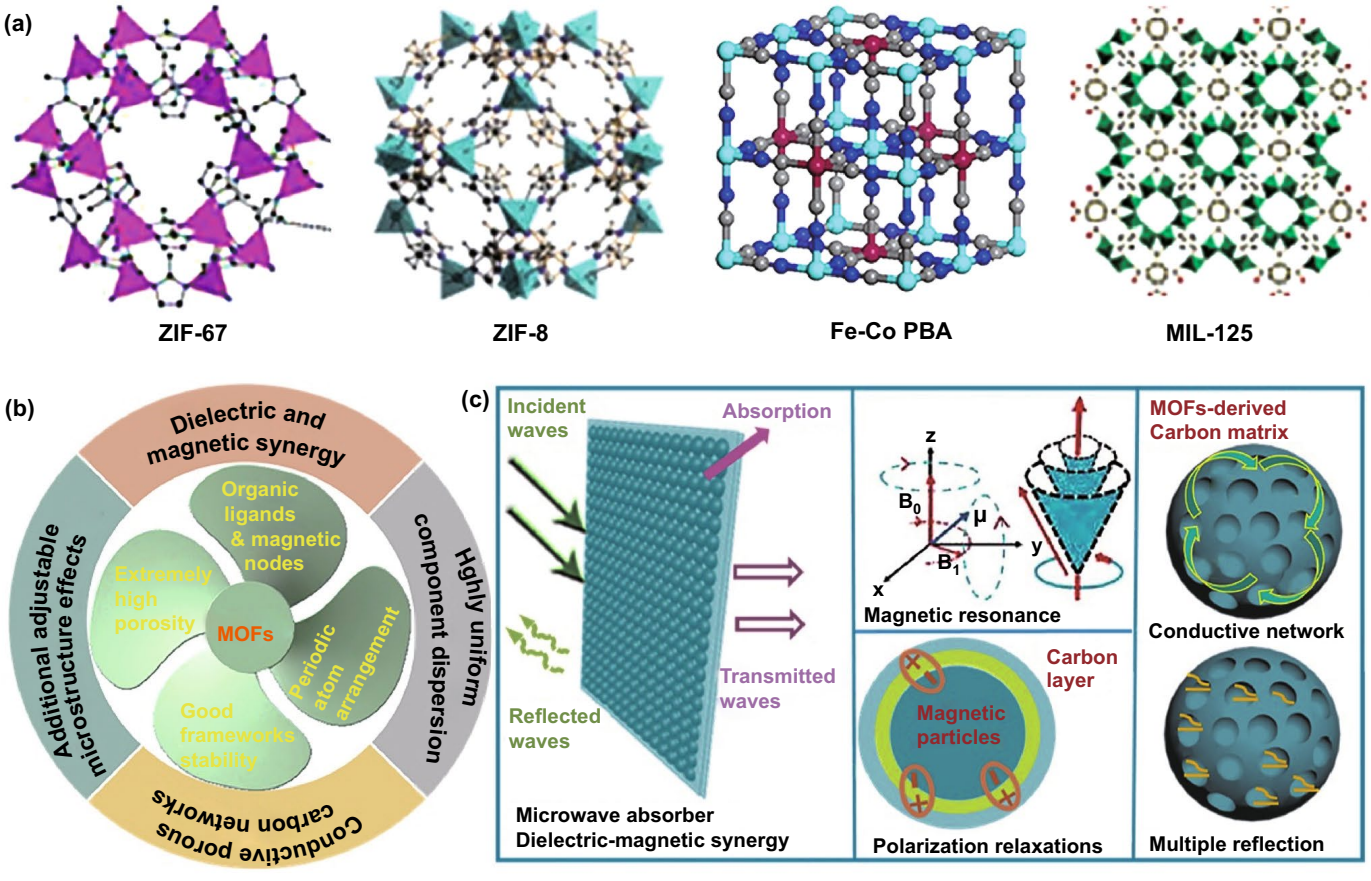
**a** Crystalline structures of some specific MOFs. Reproduced with permission from Refs. [24–27]. Copyright © 2017 WILEY–VCH; 2014 The Royal Society of Chemistry; 2018 American Chemical Society; and 2017 Wiley–VCH Verlag GmbH. **b** Some advantages of MOFs applied in the field of EM absorption, and c Electromagnetic loss and energy conversion mechanism inside MOFs-derived magnetic carbon-based composites

Metal–organic frameworks (MOFs) are a class of crystalline porous materials consisting of metal nodes joined together by organic ligands through strong coordination bonds [20]. Since the first discovery of MOF-5 with three-dimensional open skeleton structure, MOFs have attracted wide attention from academia worldwide and are considered to have great potential in catalysis, adsorption and separation, hydrogen storage [21, 22]. With the continuous development of MOFs-related fields, they are also proposed to be excellent precursors for various carbon-based functional materials due to the pyrolysis of organic ligands under high-temperature inert atmosphere [23]. Up to now, some common MOFs families, such as zeolitic imidazolate frameworks (ZIFs), Prussian blue (PB) and Prussian blue analogues (PBAs), Material Institute Lavoisier (MIL), Universitetet i Oslo (UiO), and Ni-BTC (BTC = benzene-1,3,5-tricarboxylate), have been transformed into various carbon-based materials successfully [24–28], and the crystalline structures of some specific MOFs are illustrated in Fig. 1a. Recent progress indicates that MOFs have many fascinating features that may render them as splendid sacrificing precursors for high-performance microwave absorbers (Fig. 1b, c) [29–32]. First, the widespread utilization of magnetic nodes in various MOFs makes it very easy to generate magnetic carbon-based composites, because magnetic ions will be reduced into magnetic metals through high-temperature carbothermic reduction. Carbon frameworks and magnetic metal nanoparticles will produce synergetic dielectric and magnetic loss mechanisms. Second, the periodic arrangement of different atoms in crystalline MOFs provides a congenital advantage for uniform component dispersion, and the resultant magnetic metal nanoparticles can be homogeneously decorated on MOFs-derived carbon frameworks. This situation is very favorable for full exploitation of magnetic function. Third, MOFs usually have good structure stability, which promises desirable integrity of final carbon frameworks. It is well known that carbon materials are conductive to some extent, and an intact carbon framework over several hundreds of nanometers can induce the formation of numerous microcurrent under an applied EM field, thus resulting in the enhancement of conductive loss, one main pathway of dielectric loss. Fourth, MOFs are a typical kind of porous materials, and very importantly, their extremely high porosity can be excitingly preserved in final carbon-based composites, which brings additional microstructure effects for EM attenuation, including multiple reflections and scatterings. Our group ever pioneered the synthesis of uniform Fe/C composites through a direct pyrolysis of PB nanocubes, and the high dispersion of Fe nanoparticles was found to be favorable for multiple dielectric and magnetic resonances, resulting in an excellent EM absorption performance with broad response bandwidth [31]. Lv et al. employed ZIF-67 as the precursor to produce porous Co/C nanocomposites with reflection loss (*RL*) intensity of − 35.3 dB and effective absorption bandwidth (EAB) of 5.8 GHz with the absorber thickness of 4.0 and 2.5 mm, respectively [29]. To date, there have been hundreds of papers concerned on EM absorption of MOFs-derived magnetic carbon-based composites, and most microwave absorbers therein did make some significant achievements as compared with those counterparts from conventional routes [29, 31, 33–40]. Despite of that, these MOFs-derived magnetic carbon-based composites still suffer from some undesirable drawbacks. The first one is that the final chemical compositions of these composites are highly dependent on their precursors, because carbon frameworks and magnetic metal nanoparticles are derived from organic ligands and coordination sites, respectively. Although they can display dielectric and magnetic loss capabilities as we expected, the synergy between dielectric loss and magnetic loss may not reach the best level. The second one is that the pyrolysis of MOFs usually induces the formation of microporous structure, but fails to breed some more lucrative microstructures, such as hollow, yolk-shell, multi-chamber configurations [37, 39]. These facts suggest that there is still room for the improvement on EM absorption of MOFs-derived magnetic carbon-based composites. As a matter of fact, some researchers have been aware of this aspect and devoted their efforts to elaborate composition and microstructure design in MOFs-derived magnetic carbon-based composites. In this context, we highlight some very recent advances on how to make a solid contribution to EM absorption enhancement, and we also propose some disadvantages, challenges, and prospects in this field.

## EM Absorption Mechanism, Performance Evaluation, and Influence Factors

### EM Absorption Mechanism

As we discussed above, the basic principle of EM absorption is to dissipate EM energy through the interruption of electric or magnetic field branch, and thus, dielectric loss and magnetic loss are widely considered to be two dominant mechanisms for EM absorption [31]. Dielectric loss usually originates from conductivity loss and polarization loss, where conductivity loss realizes energy consumption through the directional movement of some residual carriers in dielectric medium driven by an applied electric field [41]. Compared with conductive loss, polarization loss has more diversified modes, namely, electronic polarization, ionic polarization, dipole orientation polarization, and interfacial polarization [7]. However, ionic polarization and electronic polarization do not work the attenuation of EM waves in gigahertz range due to their extremely short relaxation time (10^–12^–10^–16^ s) and elastic nature, that is, dipole orientation polarization and interfacial polarization are active to afford the consumption of EM energy in most cases. Dipole orientation polarization is induced by the hysteretic reorientation of dipoles along with an applied electric field, and interfacial polarization depends on the asymmetrical accumulation of space charges at heterogeneous interfaces, which can generate an electric dipole moment and bring energy consumption [41]. In view of above facts, high conductivity, abundant electric diploes, and sufficient heterogeneous interfaces are very conductive to strong dielectric loss. Dielectric loss ability is usually deduced by dielectric tangent, the ratio of imaginary part to real part of relative complex permittivity $\left( {\varepsilon_{{\text{r}}} = \varepsilon^{\prime}_{{\text{r}}} - {\text{j}}\varepsilon^{\prime\prime}_{{\text{r}}} } \right)$,

1 $${\text{tan}}\delta_{{\text{e}}} = \varepsilon_{{\text{r}}}^{\prime \prime}/\varepsilon_{{\text{r}}}^{\prime}$$

Magnetic loss generally results from magnetic hysteresis, domain wall resonance, natural ferromagnetic resonance, and eddy current effect [6]. Among them, magnetic hysteresis and domain wall resonance can be easily excluded, because they are negligible in weak field and gigahertz range [9]. Natural ferromagnetic resonance directly describes energy absorption of ferromagnetic materials with large magnetization under an external anisotropy magnetic field. Once there is the formation of natural ferromagnetic resonance, there will be some typical resonance peaks in the curves of real part (*µ*_r_′) and imaginary part (*µ*_r_″) curves of relative complex permeability (*µ*_r_ = *µ*_r_′−j*µ*_r_″), and the corresponding natural resonance frequency is greatly dependent on the anisotropy energy of different magnetic particles [42]. For a given ferromagnetic material, if the natural resonance frequency shifts to a higher reign, its *µ*_r_ values will inevitably decrease, resulting in the weakening of magnetic loss ability, and this phenomenon is known as the Snoek’s limit [43]. As for eddy current effect, it is mainly caused by the thermal effect of a current along a closed circuit in a magnetic conductor [9]. It is widely accepted that if magnetic loss only derives from eddy current effect, the values of *C*_0_ (*C*_0_ = *μ*_r_″(*μ*_r_′)^−2^*f*^−1^, *f* refers to the frequency of EM waves) will keep constant and independent on the frequency [5]. Magnetic loss ability can also be deduced by magnetic tangent, the ratio of *µ*_r_'' to *µ*_r_', as shown in the following equation:

2 $${\text{tan}}\delta_{\mu } = \mu_{{\text{r}}}^{\prime \prime}/\mu_{{\text{r}}}^{\prime}$$

### Performance Evaluation

Microwave absorbers are a kind of functional materials that can realize energy conversion of incident EM waves, whose performance is the premise and cornerstone for their practical application. With the EM parameters (*ε*_r_ and *µ*_r_) mentioned above, *RL* characteristics of microwave absorbers, which directly reflect their EM absorption performance, can be easily calculated through the following equations [2]:

3 $$RL\left( {{\text{dB}}} \right) = 20\log \left| {\frac{{z_{{{\text{in}}}} - 1}}{{z_{{{\text{in}}}} + 1}}} \right|$$

4 $$Z_{{{\text{in}}}} = \sqrt {\frac{{\mu_{{\text{r}}} }}{{\varepsilon_{{\text{r}}} }}} \tanh \left[ {j\left( {\frac{2\pi }{c}} \right)fd\sqrt {\mu_{{\text{r}}} \varepsilon_{{\text{r}}} } } \right]$$

where *Z*_in_ is the normalized input impedance of microwave absorbers with a full-reflection metal substrate, *c* is the velocity of EM waves in free space (3 × 10^8^ m s^−1^), and *d* is the applied thickness of microwave absorbers. *RL* describes the absorption efficiency of incident EM waves under a given condition, and the smaller its value, the higher the absorption efficiency. When *RL* value reaches − 10.0 dB, it means 90% of incident EM waves can be effectively attenuated. The corresponding frequency region (*RL* ≤ − 10 dB) is usually defined as EAB, which is another important evaluation indicator associated with EM absorption performance [11]. Currently, the main studied frequency for microwave absorption is focused on the S-band (2*.*0–4.0 GHz), C-band (4.0–8.0 GHz), X-band (8.0–12.0 GHz) and Ku-band (12.0–18.0 GHz). To cater the demand for a specified microwave band, selective-frequency absorption is another concerned concept during the fabrication of high-performance microwave absorbers [44]. In addition to strong *RL* intensity and broad EAB, low density and thin thickness of advanced microwave absorbers are urgently pursued to determine the final practical application in modern civil and military fields.

### Influence Factors

According to EM absorption theory, the performance of microwave absorbers mainly depends on their EM attenuation capability and impedance matching condition [2]. Attenuation constant (*a*) is a parameter that can directly characterize the intrinsic loss ability of microwave absorbers in essence, and its value can be calculated by the following equation [7]:

5 $$\alpha = \frac{\sqrt 2 \pi f}{c}\sqrt {\left( {\mu^{\prime\prime}_{{\text{r}}} \varepsilon^{\prime\prime}_{{\text{r}}} - \mu^{\prime}_{{\text{r}}} \varepsilon^{\prime}_{{\text{r}}} } \right) + \sqrt {\left( {\mu^{\prime\prime}_{{\text{r}}} \varepsilon^{\prime\prime}_{{\text{r}}} - \mu^{\prime}_{{\text{r}}} \varepsilon^{\prime}_{{\text{r}}} } \right)^{2} + \left( {\mu^{\prime}_{{\text{r}}} \varepsilon^{\prime\prime}_{{\text{r}}} + \mu^{\prime\prime}_{{\text{r}}} \varepsilon^{\prime}_{{\text{r}}} } \right)^{2} } }$$

Theoretically, a larger *a* value will promise a good EM absorption performance, including minimum *RL* intensity and broad EAB. However, many previous studies have demonstrated that EM absorption performance could not be directly speculated from attenuation capability of microwave absorbers, because impedance matching is another important concept that must be taken into account seriously. A well-matched impedance can allow incident EM waves to be transmitted into the interior of microwave absorbers as much as possible, which establishes a good foundation for the consumption of EM energy [2]. The ideal condition for perfect impedance matching requires that the wave impedance of microwave absorbers is close to that of free space, and in other words, *ε*_r_ and *μ*_r_ values of microwave absorbers need to be almost identical [7]. In fact, *ε*_r_ value is usually much larger than *μ*_r_ value in most microwave absorbers, especially for many magnetic carbon-based composites, which means that the gap between *ε*_r_ and *μ*_r_ should be tailored within a rational range by artificially manipulating dielectric and magnetic components in order to fulfill good impedance matching and attenuation capability simultaneously.

It is well known that EM absorption performance is not only determined by the intrinsic EM properties of microwave absorbers, but also associated with their size, morphology/shape, and microstructure. For examples, some researchers found when the size of microwave absorbers was minimized into nanoscale, EM interaction tended to increase [45]. On the one hand, nanoscale materials usually have large specific surface area, and thus, there will be a large number of active sites on their surface, which can enhance polarization relaxation loss [46]. On the other hand, owing to high conductivity of some magnetic metals, the strong eddy current may induce undesirable skin effect, resulting in the partial invalidation of internal magnetic field and consequently decreased *μ*_r_ [29, 31]. If the particle size is smaller than the skin depth, the skin effect can be restrained and EM absorption performance of microwave absorbers will be enhanced through improved magnetic loss ability. In addition to size effect, shape/morphology and microstructure of microwave absorbers are also important factors for EM absorption, because they can affect the transmission path of incident EM waves through multiple scatterings and reflections [47]. Each scattering or reflection can bring energy loss to a certain degree, and the change in transmission behavior is equivalent to extending the transmission distance of incident EM wave in microwave absorbers, thus making considerable contribution to the conversion of EM energy [48]. Meanwhile, the manipulation on shape/morphology and microstructure is also favorable to creating more heterogeneous interfaces, which is necessary to generate powerful interfacial polarization [49]. Although there is still no clear structure–activity relationship between EM absorption performance and shape/morphology/microstructure, their positive effects have been witnessed in many published papers [47, 50], and thus, it is easy to conclude that a reasonable design on morphology/shape and microstructure of nano-scale composites is an effective strategy to develop high-performance microwave absorbers.

## Composition Optimization in MOFs-derived Magnetic Carbon-based Microwave Absorbers

As we discussed above, MOFs transformation has become a simple and effective method to produce magnetic carbon-based microwave absorbers directly, while the constant ratio of organic ligand to coordination site in each kind of MOFs sets up some obstacles to consolidate the performance of final products through composition regulation. Recent progress suggests that some special strategies have been developed to optimize the chemical compositions of MOFs-derived carbon-based microwave absorbers, including the pyrolysis of bi-metallic MOFs and the involvement of additional magnetic or carbonaceous components, as well as other dielectric components. We will introduce some typical design concepts, research findings, and performance breakthroughs carefully in the section.

### Pyrolysis of Bi-metallic MOFs

Bi-metallic MOFs contain two different inorganic metal nodes that have similar coordination activities with the same organic ligands, which offer a possibility to tailor the chemical composition of final products if one of them can be removed through high-temperature pyrolysis. It is well known that metallic Zn nanoparticles have low boiling point (600–900 °C) [51], and thus, Zn-containing bi-metallic MOFs are popular precursors in this strategy [52]. Wang et al. employed Zn–Co ZIFs as the precursor of Co/C composites, and they raised carbon content from 47.4 to 56.7 wt% by intensifying the removal of Zn-related species at high temperature [53]. When the pyrolysis temperature was 600 °C, *RL* intensity and EAB of the corresponding Co/C composite were − 50.7 dB and 4.2 GHz, respectively, with the absorber thickness of 2.5 mm. Similarly, Ji’s group introduced Zn^2+^ into Fe-MOF-5, while they found that the weight ratio of Fe nanoparticles to carbon frameworks was not changed obviously in the temperature range of 600–700 °C [54]. Although higher pyrolysis temperature resulted in a great impact on the chemical composition of final Fe/C composite, this variation weakened its EM absorption performance to a large extent because the impedance matching was much deteriorated. Subsequently, they manipulated Zn^2+^/Co^2+^ atomic ratio in bi-metallic ZIFs from 0 to 3.0 and realized the adjustment of carbon content from 38.3 to 48.7 wt%, and then harvest the strong *RL* intensity of -32.4 dB and the broadest EAB of 5.27 GHz with an absorber thickness of 1.9 mm [55]. From the current results, one can conclude that the relative content of MOFs-derived magnetic carbon-based microwave absorbers may be indeed controlled by the introduction of Zn^2+^, while the regulated range is not as obvious as expected. What’s worse, the formation of intermediate phase (i.e., Co_3_ZnC) cannot be avoided and the removal of Zn-related species is always incomplete, and thus, this method decreases the content of magnetic nanoparticles monotonously, as well as their contribution to magnetic loss.

**Fig. 2:**
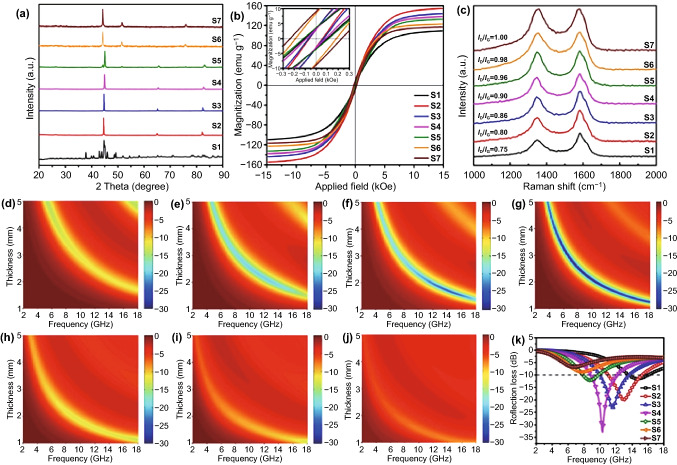
**a** XRD patterns, **b** field-dependent magnetization curves (inset is a magnification of magnetic hysteresis loops), **c** Raman spectra of S1-S7, and RL maps of **d** S1,** e** S2, **f** S3, **g** S4, **h** S5, **i** S6, **j** S7, and **k** their RL curves with the absorber thickness of 2.0 mm. Reproduced with permission from Ref. [63]. Copyright © 2018 Elsevier Inc

In order to make full use of inorganic metal sites, MOFs with dual magnetic nodes appear as more promising precursors for magnetic carbon-based composites [56–61], because the formation of alloy particles will bring additional electron transfer and spin polarizability that are also favorable for EM absorption [62]. Our group obtained a series of PB and PB analogues (PBAs) with different Co/Fe atomic ratios using a co-precipitation method and further converted them into FeCo alloy/carbon composites through high-temperature pyrolysis [63]. The characterization results revealed several advantages of dual magnetic nodes clearly. First, the existence of Co atoms, if the amount was small (Co/Fe = 0.1), could suppress the formation of Fe_3_C particles (Fig. 2a), a typical commensal of Fe particles under carbon-rich conditions, that usually put down magnetic response and magnetic loss of Fe particles (Fig. 2b) [64, 65]. Second, Co atoms had better catalytic graphitization effect than Fe atoms, and thus, more Co atoms could increase the relative graphitization degree of carbon frameworks (Fig. 2c), which was equivalent to the manipulation of carbon content [66]. Third, the involvement of Co atoms decreased the average size of magnetic particles significantly, creating more heterogeneous interfaces and more powerful interfacial polarization. These visible impacts on physicochemical properties demonstrated that EM absorption performance of FeCo alloy/carbon composites could be easily regulated by Co/Fe ratio (Fig. 2d–k). Liang et al. prepared stacked Co_*x*_Ni_*y*_@C nanosheets with Co–Ni bi-metallic MOFs as the precursor, and they also confirmed that the atomic ratio was very important for dielectric and magnetic properties of magnetic carbon-based composites [67]. When Co/Ni ratio was close to 1.0, the resultant composite could possess the best EM absorption performance, including strong *RL* intensity (− 43.7 dB) and broad EAB (5.70 GHz) with the absorber thickness less than 2.0 mm. More recently, Ouyang et al. further addressed the transformation of tri-metallic MOFs (FeCoNi-MOF-74) [68]. After pyrolysis at proper temperature, FeCoNi@C nanocomposite could reinforce *RL* intensity and EAB to − 59.0 dB and 6.4 GHz, respectively, with absorber thickness of 2.1 mm.

### Introduction of Additional Magnetic Components

Although the direct pyrolysis of bi-metallic MOFs has demonstrated its potential in the composition manipulation, it only works in a very small range that cannot meet the design requirements to improve EM properties significantly. Therefore, some groups attempted to fabricate MOFs-based composites by introducing some additional EM components and then converted these composites into magnetic carbon-based microwave absorbers with desirable chemical composition [69, 70]. By considering that magnetic loss capability in some MOFs-derived carbon-based composites is usually insufficient, the involvement of additional magnetic components becomes one effective pathway to further reinforce the overall EM properties of these composites [71]. Wang et al. introduced Co nanoparticles during the growth of ZIF-67 crystals, and they did raise the saturation magnetization and magnetic loss capability as compared with Co/C composite from pristine ZIF-67 [29, 72]. It was unfortunate that the strong interaction among Co nanoparticles caused their serious aggregation and lost the advantages of MOFs transformation [72]. Similarly, some ferrite nanoparticles, i.e., Fe_3_O_4_ and Ba_0.85_Sm_0.15_Co_2_Fe_16_O_27_, were also utilized to combine with ZIF-67 [60, 73]. The formation of FeCo alloy nanoparticles during high-temperature pyrolysis was confirmed to be greatly helpful to consolidate magnetic loss of the final composites, especially in the middle- and high-frequency range. As a result, the final FeCo/C composites could perform comparable or superior *RL* characteristics to ZIF-67-derived Co/C composites with a smaller absorber thickness. Besides, Wang et al. employed Co(OH)_2_ as the additional magnetic precursor to support ZIF-67 crystals, while they found Co(OH)_2_ could not be converted into Co nanoparticles completely even if the temperature reached 650 °C [74]. This drawback discounted the contribution of magnetic loss, and thus, Co/C composite from this way failed to produce better EM absorption performance.

**Fig. 3:**
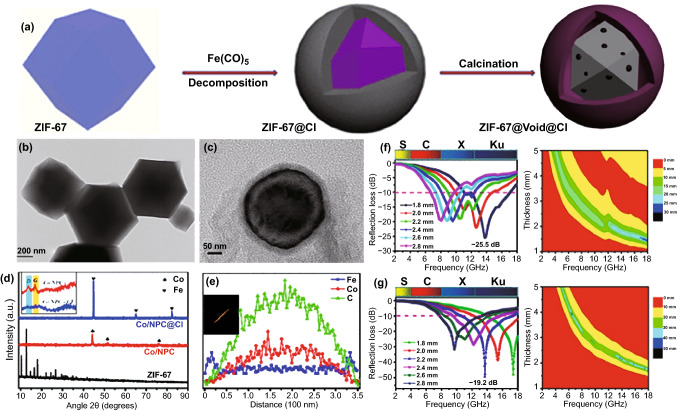
**a** Synthetic Scheme for the Preparation of Co/NPC@Void@CI, TEM images of **b** ZIF-67 and **c** Co/NPC@Void@CI, and **d** XRD patterns of samples (inset shows the Raman spectra of Co/NPC and Co/NPC@Void@CI. **e** EDS line scans of Co/NPC@Void@CI, RL maps and RL curves of **f** Co/NPC and **g** Co/NPC@Void@CI. Reproduced with permission from Ref. [77]. Copyright © 2017 American Chemical Society

Beyond the direct introduction of magnetic metal and ferrite nanoparticles, some elaborate strategies were further developed in recent years. For example, Yan et al. and Wang et al. soaked Zn-Co bi-metallic MOFs in Fe^3+^ solution, and both of these two groups found that the impregnation of Fe^3+^ could be favorable for the generation of FeCo alloy nanoparticles and the removal of Zn species under high temperature could create desirable porosity [75, 76]. When the atomic ratio of Fe to Co was 0.26, the resultant FeCo/Co/C composite displayed the strongest *RL* intensity over − 60.0 dB and the corresponding EAB as broad as 5.1 GHz with the absorber thickness of 1.5 mm [76]. Quan et al. conducted the decomposition of Fe(CO)_5_ on the surface of ZIF-67 and then harvested nanoporous carbon-wrapped Co@carbonyl iron with yolk-shell structure (Co/NPC@Void@CI) through high-temperature pyrolysis (Fig. 3a) [77]. TEM images recorded the microstructure evolution from ZIF-67 to Co/NPC@Void@CI (Fig. 3b, c). XRD patterns and EDS line scans confirmed the formation of carbonyl iron on the external surface of Co/NPC (Fig. 3d, e). More importantly, the EM absorption performance of Co/NPC@Void@CI was greatly superior to that of Co/NPC (Fig. 3f, g), whose strongest *RL* intensity and EAB were − 49.2 dB and 6.72 GHz, respectively, with the absorber thickness of 2.2 mm.

### Introduction of Additional Carbon Components

Compared with various magnetic components, the introduction of additional carbon components into MOFs-derived composites receives much more attention, because carbon materials have morphological diversity as well as better MOFs compatibility than magnetic metals or ferrites. Yang et al. deposited NiFe PBAs nanocubes on graphene oxide uniformly to be the precursor of NiFe@C/reduced GO (rGO) composite [78]. With the adjustment of pyrolysis temperature, the optimal NiFe@C/rGO composite displayed strong *RL* intensity close to − 40.0 dB and high absorption efficiency (over 90%) in X band. Inspired by this work, rGO becomes a popular candidate to regulate EM properties of MOFs-derived magnetic carbon-based composites [79–82]. The introduction of rGO not only reinforces the overall dielectric loss of carbon-based composites, but also provides powerful polarization relaxation through more heterogeneous interfaces. It is worth noting that the advantages of bi-metallic MOFs are highly considered when MOFs/GO precursors are fabricated. For example, Xu et al. assembled CoNi-1,3,5-trimesic acid microspheres and GO nanosheets to produce CoNi@NC/rGO comprising pomegranate-like CoNi@NC nanoclusters and ultrasmall CoNi-decorated graphene [83]. EM measurements manifested that CoNi@NC/rGO could be a promising microwave absorber with strong *RL* intensity (− 68.0 dB) and broad EAB (6.72 GHz) with the absorber thickness of 3.0 and 2.5 mm, respectively, and its performance was greatly superior to those of individual CoNi@NC or CoNi/rGO. Zhao et al. also demonstrated the positive contribution of alloy nanoparticles and rGO nanosheets clearly, where CoNi@NCPs-rGO could lower minimum *RL* intensity of Co@NCPs from − 49.8 to − 58.2 dB [84]. Very interestingly, Wang et al. decorated bi-metallic FeCo-ZIFs on the surface of freeze-drying rGO aerogel and then converted the precursor into porous cocoon-like FeCo/NC/rGO composite [85]. They found that this composite possessed many desirable characteristics favorable for EM absorption, such as magnetic loss, dielectric loss, resistance loss, interfacial polarization, and good impedance matching, which were responsible for its very broad EAB of 9.29 GHz with the absorber thickness of 2.63 mm. In some cases, metal sites in MOFs were transformed into magnetic ferrites at relatively low temperature, while the presence of rGO nanosheets could also optimize their EM properties and bring considerable EM absorption performance [86].

**Fig. 4:**
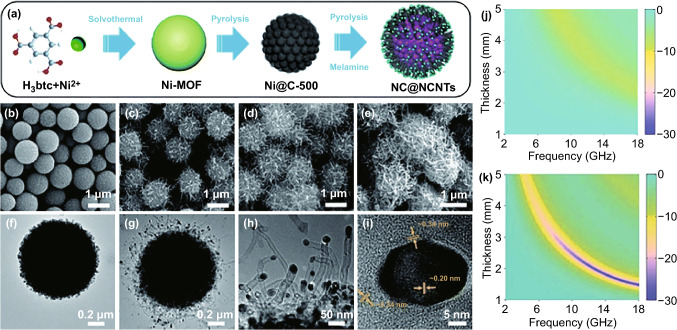
**a** Schematic of the preparation of the NC@NCNTs. SEM images of **b** NC, **c** NC@NCNTs-1, **d** NC@NCNTs-2, and **e** NC@NCNTs-3. TEM images of **f** NC and **g-i** NC@NCNTs-2. RL maps of **j** NC, **k** NC@NCNTs-2. Reproduced with permission from Ref. [96]. Copyright © 2021 The Royal Society of Chemistry

Moreover, there is also growing interest in the composition regulation of MOFs-derived magnetic carbon-based composites with carbon nanotubes (CNTs) and carbon nanofibers (CNFs) instead of rGO nanosheets, because their one-dimensional configuration will be beneficial to the formation of conductive networks in resin matrix and consequently intensify the dielectric loss of carbon-based composites [87–93]. However, the mismatch between the diameter of 1D carbon materials and the particle size of MOFs microspheres/polyhedrons makes it very difficult to realize their homogeneous combination. This situation offsets the advantages of MOFs-derived magnetic carbon-based microwave absorbers to a certain extent. In situ growth of CNTs on the surface of MOFs-derived magnetic carbon-based composites appears as an effective way to remediate their poor chemical homogeneity [94, 95]. Our group employed waxberry-like Ni@C microspheres (NC) derived from Ni-MOFs as the substrate and induced the growth of CNTs by feeding melamine vapor (Fig. 4a) [96]. The amount of CNTs was verified to be highly dependent on the mass ratio of melamine to Ni@C microspheres (Fig. 4b–e). Ni nanoparticles on the surface of Ni@C microspheres played an important role to conduct CNTs growth through a “vapor–liquid–solid” mechanism, and thus, there were many Ni nanoparticles encapsulated in the tips of CNTs (Fig. 4f–i). The relative carbon content of final composites (NC@NCNTs) could be easily tailored from 23.0 to 60.3 wt% just by manipulating the mass ratio of melamine to Ni@C microspheres from 0 to 10. When the relative carbon content was 51.1 wt%, NC@NCNTs proclaimed its better impedance matching and EM absorption than pristine NC even in the lower filler loading (Fig. 4j–k) [40, 86]. Liu et al. recently found that glucose could also be utilized additional carbon source [97]. They soaked Co_3_[HCOO]_6_·DMF in ethanol solution with glucose, while the content of final Co/C composites would be less changed until the mass ratio of glucose to Co_3_[HCOO]_6_·DMF reached 5.0. In our latest research, we innovatively revealed dual functions of glucose in isopropyl alcohol [9]. On the one hand, it would be the source of gluconate as the organic ligand to complex with Co ions and produce uniform Co-MOFs microspheres, and on the other hand, it could be converted into carbon nanoparticles and accommodated in Co-MOFs microspheres. With these functions of glucose, we improved the morphology of Co/C composites and optimized their EM properties. The best candidate among this series of composite displayed very strong *RL* intensity of − 71.3 dB and its EAB could cover the frequency range of 3.5–18.0 GHz by manipulating the absorber thickness of 1.0–5.0 mm. This study provided a new idea to regulate the chemical composition of MOFs-derived magnetic carbon-based microwave absorbers.

### Introduction of Other Dielectric Components

**Fig. 5:**
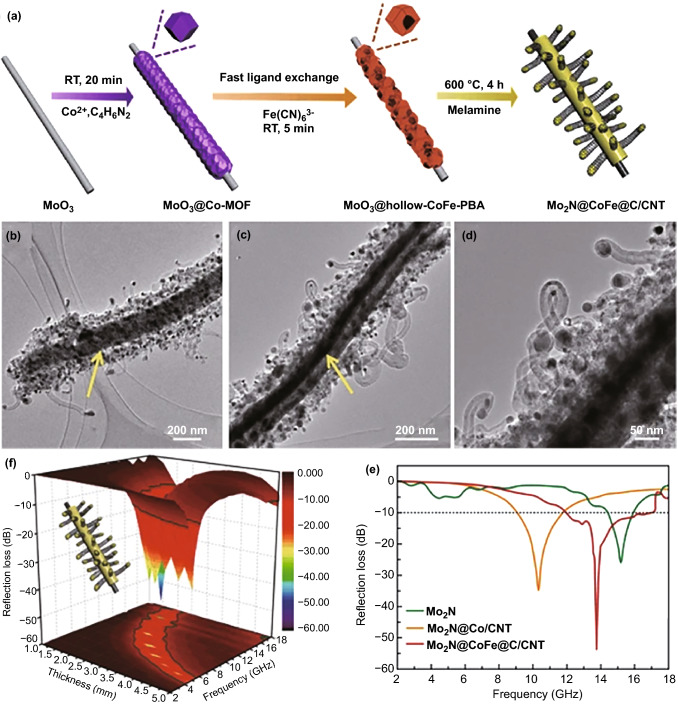
**a** Schematic process of the fast MOF-based ligand exchange strategy for construction of 3D hierarchical Mo2N@CoFe@C/CNT composites. **b**–**d** TEM images of Mo2N@CoFe@C/CNT composites, and **e** 3D RL map, **f** RL curves of Mo2N@CoFe@C/CNT at the same thickness of 2 mm. Reproduced with permission from Ref. [105]. Copyright © 2021 Springer

Apart from additional magnetic and carbon components, some other dielectric components, such as metal oxides, carbides, and conductive polymers, can also be involved into MOFs-derived composites to consolidate their intrinsic EM loss capabilities. Metal oxides are one of the most popular additional components to couple with magnetic carbon-based composites due to their easy preparation and chemical stability, as well as their effective improvement on impedance matching. In general, metal oxides, e.g., TiO_2_ and MnO_2_, are preferentially deposited on the external of MOFs [98, 99]. After high-temperature pyrolysis, some ternary composites with typical core–shell configuration can be obtained. Such a delicate design not only enables the synergistic effects between magnetic loss from metal nanoparticles and intrinsic dielectric losses from carbon frameworks and MnO_2_ shells, but also facilitates multiple interface polarization and appropriate impedance matching. Wang et al. further mediated the growth of MnO_2_ nanosheets on the surface of ZIF-67 nanocubes with a polydopamine (PDA) layer, which could also be transformed into carbon layer after high-temperature pyrolysis [100]. This work demonstrated how to manipulate dielectric loss of MOF-derived magnetic carbon-based composites from two aspects. The final Co/C@MnO_2_ composite could exhibit good EM absorption performance with the minimum *RL* intensity of − 58.9 dB and decent EAB of 5.5 GHz, with the absorber thickness of 3.7 and 1.9 mm, respectively. In contrast, some groups conducted the growth of MOFs on metal oxides in order to consolidate EM properties of carbon-based composites with their profitable morphological features [101–104]. Of note is that Xu et al. deposited ZIF-67 nanocrystals on MoO_3_ nanorods and then converted the mixture into MoO_3_@Co-Fe PBAs through fast ligand exchange, and finally, a quadruple composite of Mo_2_N@CoFe@C/CNT could be generated by pyrolyzing MoO_3_@Co-Fe PBAs in the presence of melamine (Fig. 5a) [105]. In this composite, abundant magnetic CoFe nanoparticles were encapsulated within one-dimensional graphitized carbon/carbon nanotubes supported on Mo_2_N nanorods (Fig. 5b–d). The synergic magnetic–dielectric effects and multi-dimension hierarchical configuration rendered this composite as a promising microwave absorber with strong *RL* intensity (− 53.5 dB) and broad EAB (5.0 GHz, Fig. 5e, f), with the absorber thickness of 2.0 mm.

**Fig. 6:**
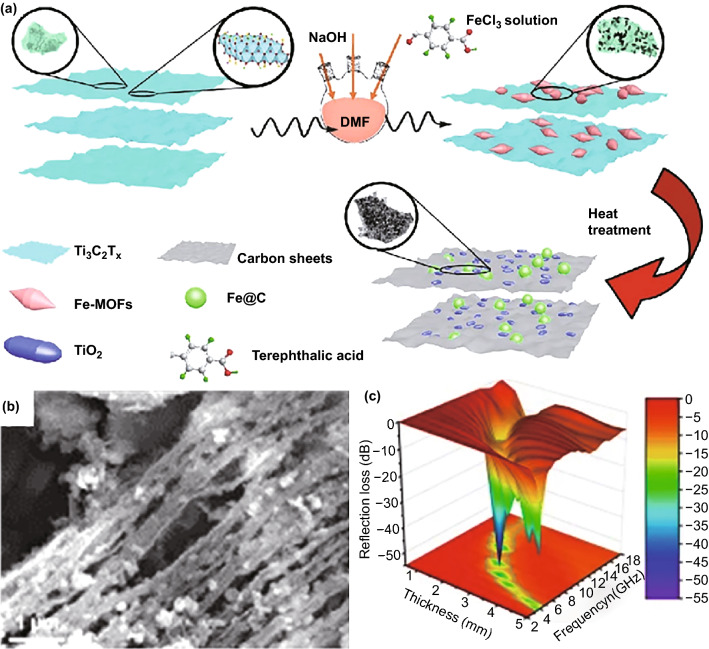
**a** Schematic representation of the facile synthesis route of the Fe&TiO2@C. **b** SEM image and **c** 3D RL map of Fe&TiO2@C. Reproduced with permission from Ref. [116]. Copyright © 2020 Springer

As typical dielectric ceramics, carbides are also usually involved in carbon-based composites due to their natural characteristics of polarization relaxation [106–109], where silicon carbide (SiC) is one of the most popular carbides in EM absorption [110–113]. Yang et al. ever attempted to integrate SiC with MOF-derived magnetic carbon-based composites, while the final SiC/Ni/NiO/C composite from the mixture of SiC nanoparticles and Ni-MOFs failed to produce acceptable EM absorption [111]. This situation may be significantly improved if SiC nanowires are applied [110]. When Co/Co_3_O_4_/C polyhedrons derived from ZIF-67 are penetrated by SiC nanowires, the minimum *RL* intensity will be pulled down beyond − 30.0 dB and EAB will be extended to 5.92 GHz with the absorber thickness of 3.0 and 2.0 mm, respectively [110]. The interconnection of SiC nanowires was considered to be important for the enhanced EM absorption performance. Two-dimensional metal carbides, MXenes, were also employed to support Fe-MOF-derived Fe_3_O_4_@C nanoparticles [114]. Although the final Fe_3_O_4_@C/Ti_3_C_2_T_*x*_ gave better performance than individual Fe_3_O_4_@C and Ti_3_C_2_T_*x*_, its EAB was only 3.5 GHz, which meant that the overall enhancement effect was not as good as that of one-dimensional SiC nanowires [110]. It is very interesting that MXenes can further exert their contribution as the only source for both carbon nanosheets and TiO_2_ nanoparticles [115]. For instance, Deng et al. filled the interspaces of MXenes nanosheets with Fe-MOFs and harvested Fe&TiO_2_@C composites by heat treatment under H_2_/Ar atmosphere [116] (Fig. 6a). Most Fe@C nanoparticles derived from Fe-MOFs and those generated in situ TiO_2_ nanoparticles were uniformly dispersed into the interspaces of carbon nanosheets from MXenes (Fig. 6b). The strongest *RL* intensity of this sandwich-like composite was − 51.8 dB, and the corresponding EAB reached 6.5 GHz (Fig. 6c). In addition to intrinsic loss capacities of Fe, TiO_2_, and C, they also attributed the good performance of Fe&TiO_2_@C composite to the capacitor‑like structure constructed by multiple components, as well as considerable polarization effect of their abundant heterogeneous interfaces. The particle sizes of conventional SiC and MXenes are usually large and randomly distributed, and thus, it is difficult to make some rational design on their related composites. In this context, Mo_2_C has been taken as a promising candidate for SiC and MXenes due to its very small particles size [106, 107]. Our group prepared ternary Mo_2_C/Co/C composites with MoO_3_ nanorods attached ZIF-67 crystals as the precursor [117]. It was revealed that dipole orientation polarization and interfacial polarization provided by small Mo_2_C nanoparticles made solid contribution to dielectric loss, and the optimized composite with 30.9% of carbon, 53.6% of Mo_2_C, and 15.5% of Co exhibited *RL* intensity over − 48.0 dB and a broad integrated EAB of 3.0–18.0 GHz by accumulating the absorber thickness. With respect to conductive polymers, they can only be involved into final magnetic carbon-based composites after MOFs transformation, and otherwise, they will be also converted into carbon components during heat treatment process [118, 119]. Sun et al. coated Co/C polyhedrons from ZIF-67 by polypyrole nanoparticles and achieved broad EAB of 6.6 GHz by manipulating the composite loading in resin matrix [120].

## Microstructure Design in MOFs-derived Magnetic Carbon-based Microwave Absorbers

It is well known that the attenuation of EM waves is not only determined by the intrinsic EM properties of microwave absorbers, but also highly associated with their interior microstructure that may promote energy consumption through multiple reflection or scattering behaviors of incident EM waves [121, 122]. As a result, there is still great interest in developing various profitable microstructures in MOFs-derived magnetic carbon-based microwave absorbers except from those naturally derived micropores and mesopores, in despite of the fact that some significant breakthroughs from composition optimization have been witnessed. In this section, we focus on several effective strategies for the construction of some unique microstructures in MOFs-derived magnetic carbon-based microwave absorbers that can promote their EM absorption performance greatly.

### Chemical Etching

**Fig. 7:**
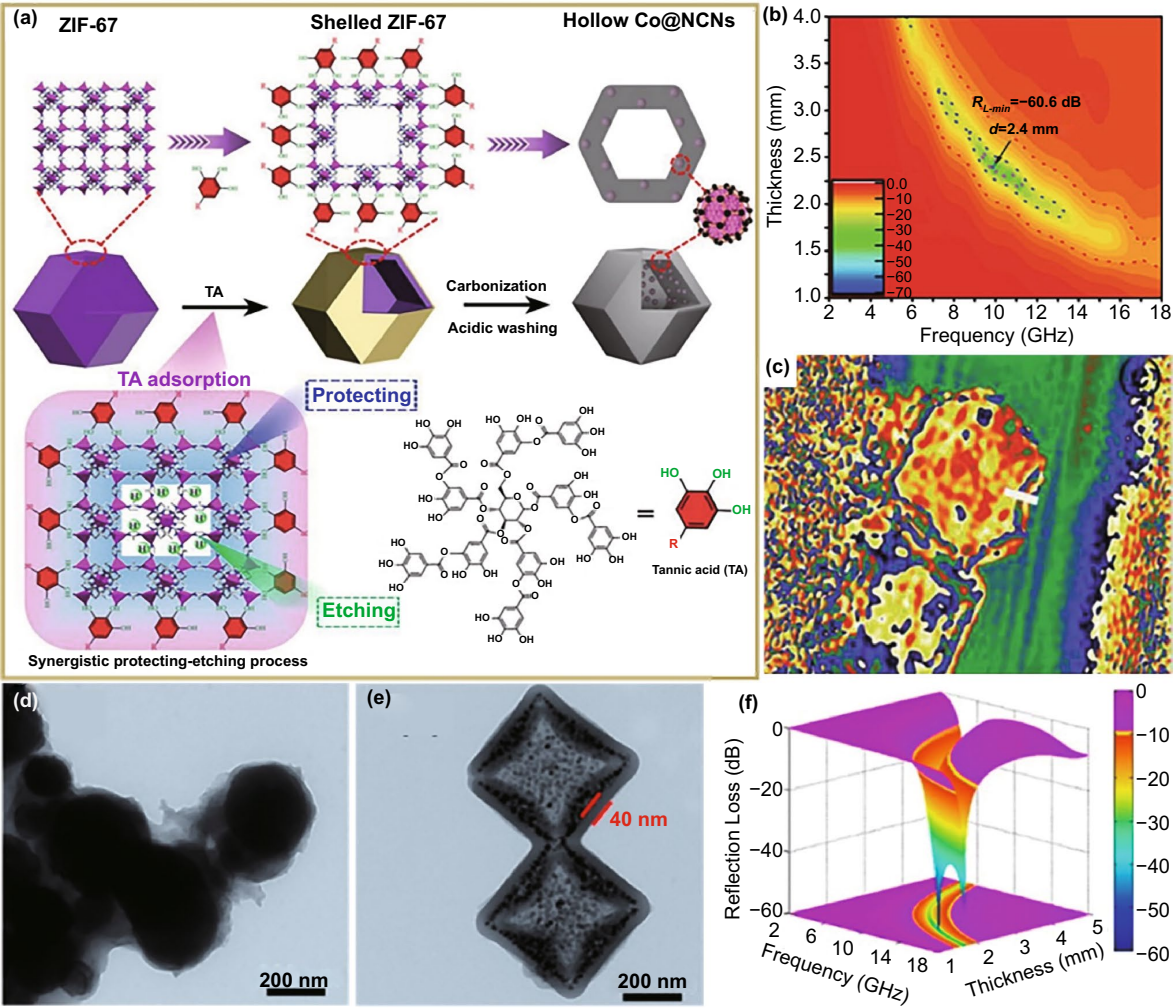
**a** Schematic illustration for the synthetic procedure of hollow Co@NCNs. **b** RL maps and **c** charge density map of hollow Co@NCNs-800*.* Reproduced with permission from Ref. [129]*.* Copyright © 2021 Wiley–VCH GmbH*.* TEM images of **d** NiCo@C-0 and **e** NiCo@C-2; **f** 3D RL map of NiCo@C-2*.* Reproduced with permission from Ref. [134]*.* Copyright © 2020 Elsevier Ltd

Chemical etching is one of the most direct methods to create hollow cavity in many functional materials [123–126]. By considering good chemical stability of carbon frameworks, it is impossible to carry out microstructure construction in final MOFs-derived carbon-based composites, and thus, chemical etching must be rationally applied to MOFs crystals before their pyrolysis. However, in most cases, MOFs etching starts from their external surface and results in morphology evolution to some extent [123, 127, 128]. At present, there are two possibilities to cause the internal etching of MOFs crystals as we expected, which is a prerequisite to introduce hollow microstructure in final carbon-based composites. One is to break the coordination bonds between metal nodes and organic ligands through protons released from organic acids (e.g., tannic acid (TA) and gallic acid) tightly attached on the surface of MOFs crystals [129, 130]. Thanks to the strong coordination effect of organic acids with metal sites on the surface, MOFs crystals will maintain their original morphologies during the etching process [131]. In this way, Liu et al. reported the formation of hollow ZIF-67 rhombic dodecahedral cages with inner hollow cavity, where TA displayed dual functions as both protecting and etching agents (Fig. 7a) [129]. After high-temperature pyrolysis and sulfuric acid treatment, hollow Co/C cages with uniform heterojunctions were obtained, and their EM properties could be optimized when the pyrolysis temperature was 800 °C, whose minimum *RL* intensity and EAB were − 60.6 dB and 5.1 GHz with the absorber thickness of 2.4 and 1.9 mm, respectively (Fig. 7b). More importantly, the authors recorded different density states of charge distribution at the interfacial regions of carbon shell and inner cavity and identified that the unbalanced charge distribution would accumulate around the interface under the external EM field (Fig. 7c). These results witnessed that the construction of hollow microstructure was indeed helpful to induce the interfacial polarization favorable for EM absorption.

The other mode for the internal etching of MOFs crystals is established on the inhomogeneity of their crystalline structure, where the crystallographic surface with a high concentration of coordination bonds will be preferentially decomposed in the presence of etching agents [123, 132]. Han et al. [133] further proposed that the corners and edges of MOFs crystals were highly active sites to react with etching agents due to their large curvatures and high surface energies, and the etching rate along the diagonals of MOFs crystals was faster than that in other directions. We ever obtained hollow NiCo PBAs microboxes with truncated eight vertexes through ammonia etching [134]. It was unfortunate that these hollow PBAs microboxes lost their microstructure after high-temperature pyrolysis, and final NiCo@C composite only consisted of numerous agglomerated nanoparticles (Fig. 7d). By coating phenolic resin (PR) on the surface of hollow NiCo PBA microboxes before high-temperature pyrolysis, the desirable microstructure could be well retained, which demonstrated that PR layer could efficiently reinforce the thermal stability of hollow PBAs microboxes (Fig. 7e). In fact, PR layer was not only helpful for the reservation of hollow microstructure in final composites, but also optimized their chemical composition simultaneously. EM measurements revealed that optimized hollow NiCo@C microboxes produced much better EM absorption performance than NiCo@C nanoparticles, whose minimum *RL* intensity was as high as − 68.4 dB and the integrated EABs were 14.1 GHz by integrating the absorber thickness from 1.0 to 5.0 mm (Fig. 7f).

### Template-Mediated Assembly

**Fig. 8:**
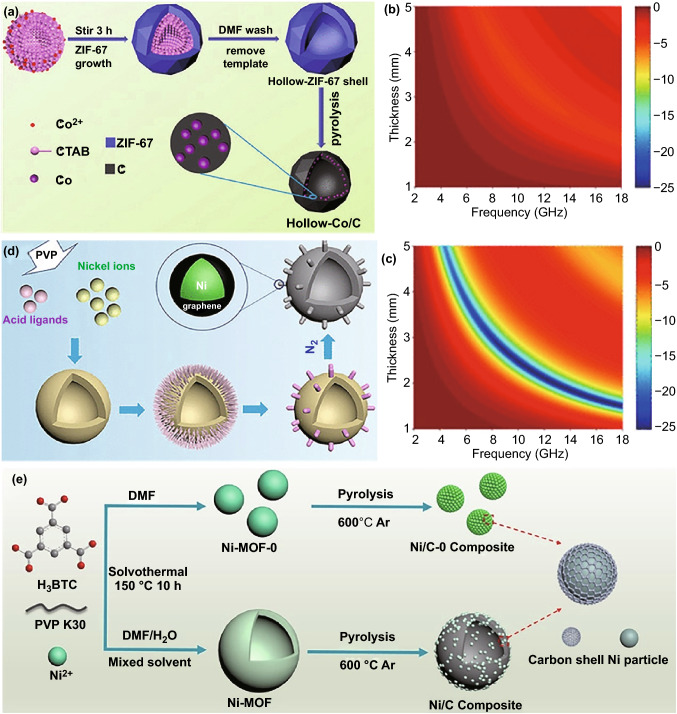
**a** Schematic illustration of preparing hollow Co/C microspheres via a MOFs-derived strategy, RL maps of **b** Co/C and **c** Co/C-HS-600. Reproduced with permission from Ref*.* [143]. Copyright © 2018 American Chemical Society*.* **d** Illustration for the formation of Ni-MOF hollow spheres with controllable surface architecture. Reproduced with permission from Ref. [145]. Copyright © 2019 American Chemical Society. **e** Schematic illustration of the formation of Ni/C composites. Reproduced with permission from Ref*.* [32]*.* Copyright © 2019 Elsevier B.V

The template-mediated strategy, including both soft template and hard template, has demonstrated its effectiveness to create some additional microstructures in some MOFs-derived carbon-based composites [135–139]. There are two critical factors for the formation of those desirable microstructures: one is the interaction between metal ions/organic ligands and templates that may direct the assemble procedure of MOFs nanocrystals, and the other is the thermal stability of intermediate MOFs that determines microstructure preservation in final carbon-based composites [60, 135, 140–142]. Our group led the way in applying this strategy for the preparation of MOFs-derived magnetic carbon-based microwave absorbers [143]. With the introduction of cetyltrimethylammonium bromide (CTAB), hollow ZIF-67 microspheres could be generated by utilizing CTAB vesicles as the nucleation sites for heterogeneous outward-growth of ZIF-67 nanocrystals (Fig. 8a). It was very exciting that final Co/C microspheres could inherit this hollow microstructure very well, and exhibit much better *RL* characteristic than Co/C composites derived from conventional ZIF-67 polyhedrons (Fig. 8b, c). However, CTAB is not a universal soft template for hollow MOFs and their derivatives, because the assembly of CTAB vesicles and ZIF-67 nanocrystals is strongly dependent on the contribution of counter ions and electrostatic interaction [144]. By comparison, polyvinyl pyrrolidone (PVP) seems to be a microstructure directing agent with good universality for different hollow MOFs and, to date, hollow Ni-1,3,5-trimesic acid microspheres (Fig. 8d, e), FeMn PBAs nanoboxes, and FeCo PBAs nanoboxes have been successfully fabricated with the assistance of PVP [32, 145, 146]. The resultant Ni/C, FeMn/C, and FeCo/C composites with typical hollow microstructure all produce better EM absorption performance than their solid counterparts.

**Fig. 9:**
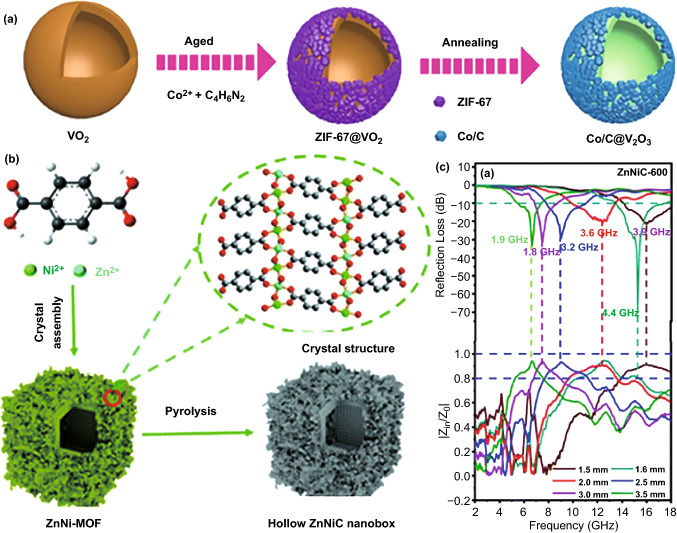
**a** Synthesis process of hierarchical Co/C@V_2_O_3_ hollow spheres. Reproduced with permission from Ref. [104]*.* Copyright © 2019 Wiley–VCH Verlag GmbH & Co. **b** Schematic illustration of hollow ZnNiC nano-box preparation*,* **c** RL curves and normalized input impedance of ZnNiC-600. Reproduced with permission from Ref. [142]*.* Copyright © 2020 The Royal Society of Chemistry

In addition, there are also some examples on microstructure design for EM absorption enhancement via a hard-template pathway. For instance, Zhou et al. introduced hollow microstructure with hollow VO_2_ microspheres as hard template and coated them with a uniform ZIF-67 layer [104, 142]. Instead of the template removal in conventional hard-template route, hollow VO_2_ microspheres were converted into hollow V_2_O_3_ microspheres in final composite and played as auxiliary dielectric components for EM attenuation (Fig. 9a). It was found that hollow microstructure not only improved impedance matching, but also intensified interfacial polarization, and as a result, the strongest *RL* intensity and EAB value of composite were − 40.1 dB and 4.6 GHz, respectively, when the absorber thickness was 1.5 mm. Miao et al. found that MOF-5 could be firstly generated in a mixed solution with zinc acetate dehydrate, nickel (II) acetylacetonate, and terephthalic acid and play as a hard template for growth of metastable bimetallic Ni–Zn MOFs (Fig. 9b) [142]. After the dissolution of internal MOF-5, hollow Ni-Zn MOFs could be obtained and transformed into hollow NiZnC nanoboxes subsequently through high-temperature pyrolysis. Similarly, the positive contribution from hollow microstructure to multiple reflection and polarization relaxation was also highlighted, which endowed hollow NiZnC nanoboxes with very strong specific *RL* intensity (41.3 dB mm^−1^, Fig. 9c).

**Fig. 10:**
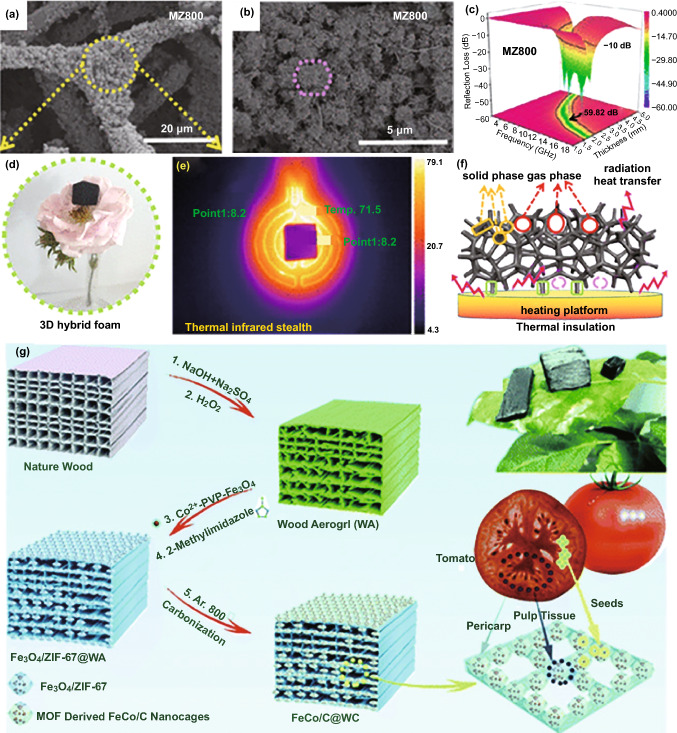
**a, b** SEM images of MZ700, **c** 3D RL map of MZ800, **d** density test of MZ800, **e** thermal infrared images of MZ800 captured at 30 min, **f** schematics of the heat transfer mechanism. Reproduced with permission from Ref. [147]*.* Copyright © 2020 American Chemical Society*. ***g** Establishment of the tomato-like hierarchical porous FeCo/C@WC. Reproduced with permission from Ref. [154]*.* Copyright © 2020 The Royal Society of Chemistry

Apart from hollow microstructure in microwave absorbers, three-dimensional macroporous microstructure has also been recognized to be greatly helpful for the consumption of EM energy [11, 41, 147, 148]. However, it is very difficult to create such a profitable microstructure just by a direct pyrolysis of MOFs. Therefore, some groups attempted to support MOFs crystals on hard templates with unique three-dimensional macroporous microstructure and integrate them into magnetic carbon-based composites during high-temperature pyrolysis. Melamine-based foams are emerging as a kind of popular MOFs scaffolds to produce three-dimensional macroporous magnetic carbon-based composites [11, 147, 149]. For example, Gu et al. manipulated the deposition of ZIF-67 on the surface of melamine foam in an ice bath and obtained three-dimensional macroporous Co/C composites (MZT) under high-temperature inert atmosphere [147]. The microstructure of melamine foam was perfectly preserved in final composites, where ZIF-67-derived Co/C polyhedrons were homogeneously distributed on carbon skeletons originated from melamine foam (Fig. 10a, b). Actually, the involvement of melamine foam also regulated carbon content of Co/C composites to some extent. These positive changes not only made a solid contribution to EM absorption in both *RL* intensity (− 59.8 dB) and EAB (5.64 GHz), as well as ultrabroad EM response in the frequency range of 2.0–18.0 GHz (Fig. 10c), but also brought some additional merits in lightweight and heat insulation (Fig. 10d, f), which rendered these Co/C composites as multi-functional materials with great potential in plenty of practical applications. Biomass materials are also common three-dimensional scaffolds in virtue of their biological structure [150, 151]. Wheat flour and cotton fiber were utilized as biomass hard templates to combine with Co/C polyhedrons derived from ZIF-67 crystals [152, 153], and both of them could promise effective improvements in EM absorption, and especially for cotton fiber, the corresponding Co/C composite strengthened *RL* intensity down to − 50.0 dB and extended EAB even to 8.0 GHz. Xiong et al. impregnated delignified wood aerogel (WA) into a stock solution of Co^2+^, PVP, and Fe_3_O_4_ nanoparticles and then incubated Fe_3_O_4_/ZIF-67@WA with the help of 2-methylimidazole and finally converted Fe_3_O_4_/ZIF-67@WA into hierarchical composite (FeCo/C@WC) with tomato-like polystage micro-nanoarchitecture (Fig. 10g) [154]. FeCo alloy nanoparticles could be uniformly dispersed on the surface of carbon skeletons from WA. The presence of the microstructure is quite beneficial to the construction of conductive network, thus facilitating electron transfer and upgrading conductivity of composites. By optimizing the loading of Fe_3_O_4_ nanoparticles, this hierarchical composite could exhibit superior EM absorption with strong *RL* intensity less than − 40.0 dB and desirable EAB as broad as 8.9 GHz with the absorber thickness of 1.5 and 1.9 mm, respectively.

### Interfacial Ion Exchange

**Fig. 11:**
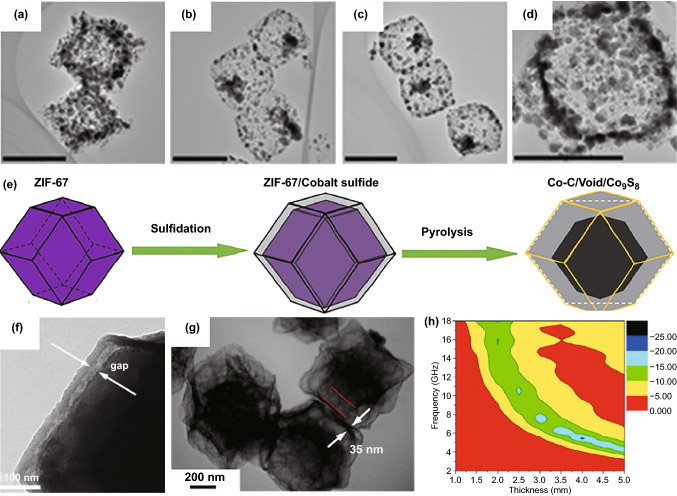
TEM images of **a** CoNi@NG-NCP-30, **b** CoNi@NG-NCP-60, **c** CoNi@NG-NCP-90, and **d** CoNi@NG-NCP-120 (scale bars in the images: 500 nm). Reproduced with permission from Ref. [158]*.* Copyright *©* 2018 American Chemical Society. **e** Synthetic processes of the Co–C/Co_9_S_8_ composite, TEM images of **f** ZIF-67/Co_9_S_8_-3 and **g** Co–C/Co_9_S_8_-3, and **h** RL map of Co–C/Co_9_S_8_-3. Reproduced with permission from Ref. [163]*.* Copyright © 2018 Springer

Interfacial ion exchange is an alternative strategy to create hollow microstructure in the absence of any soft/hard templates, where a stable heterogeneous shell will be firstly generated through the exchange of organic ligands on MOFs surface with some anionic ions in solution, and then, a hollow cavity can be shaped with the constant consumption of interior MOFs core and reserved in final carbon-based composites after high-temperature pyrolysis [155, 156]. Wang and co-workers found that nickel nitrate could be used to react with ZIF-67 to form uniform NiCo-LDH coating on the surface through a hydrolysis-controlled ion-exchange process [157]. During the reaction, the hydrolysis of Ni^2+^ produced numerous protons, which broke the chemical bond between metal center and ligand group, and the outward diffusion of cobalt ions induced the formation of a big void inside NiCo-LDH shell. Hollow carbon polyhedrons with uniform dispersion of CoNi alloy were finally obtained. EM measurements confirmed that minimum *RL* intensity and EAB with the absorber thickness of 2.0 mm of such a hollow CoNi/C composite were − 61.0 dB and 5.2 GHz, respectively. Zhang et al. further revealed that the void space between ZIF-67 core and NiCo-LDH shell could be rationally regulated by reaction time, and however, no matter when the reaction was terminated, all intermediate ZIF-67@NiCo-LDH precursors would be converted into hollow carbon polyhedrons decorated by NiCo nanoparticles (Fig. 11a–d) [158]. The only thing that changed with reaction time was Ni/Co atomic ratio. The composite with a relatively high Ni/Co ratio (0.95) also displayed broad EAB close to 5.0 GHz with the absorber thickness of 2.0 mm, and meanwhile, the introduction of Ni atoms could more or less raise oxidation resistance of magnetic metal nanoparticles therein. It was very interesting that Zhao et al. applied this strategy on the surface of short CNFs [159]. As the support of hollow magnetic carbon polyhedrons, short CNFs optimized carbon content in final composites and realized desirable EM enhancement, further broadening EAB more than 6.0 GHz. In addition to routine OH^−^, S^2−^ is also considered to be a kind of effective anions that may work for interfacial ion exchange of MOFs [160–162]. For example, when ZIF-67 was impregnated into thioacetamide solution, there would be a thin layer of cobalt sulfide due to the combination of Co^2+^ and S^2−^, and a gap between ZIF-67 core and sulfide shell could be observed because the dissolution of ZIF-67 was faster than the deposition of cobalt sulfide (Fig. 11e, f) [163]. Of note was that the final composites presented typical yolk-shell microstructure (Fig. 11g), a more favorable configuration for EM consumption [121], with Co–C as the core and Co_9_S_8_ as the shell. Multiple interfacial polarizations and multiple reflections of EM waves induced by the distinct core@void@shell architecture endowed this Co/C/Co_9_S_8_ composite with excellent EAB as broad as 8.2 GHz (Fig. 11h).

Along with the flourish of mussel-inspired surface engineering [164], PDA is evolving as a structure-directing agent to induce interfacial ion exchange, because it can provide stronger chemical complexation to metal ions than those conventional organic ligands [165]. Moreover, PDA can be also taken as a supplementary carbon source to amend EM properties of magnetic carbon-based composites [166, 167]. Wang et al. [100] demonstrated the conversion of ZIF-67 polyhedrons into hollow Co/C nanoboxes with the assistance of PDA layer. Co^2+^ released from the precipitation-dissolution equilibrium of ZIF-67 crystals would be quickly captured by PDA layer attached on ZIF-67 polyhedrons, resulting in the formation Co-PDA shells. In this case, the equilibrium was destroyed, and thus, Co^2+^ would constantly release from interior ZIF-67 cores, which caused the inward shrinkage of ZIF-67 cores and their separation with external Co-PDA shells. Finally, hollow Co-PDA nanoboxes were yielded and further transformed into hollow Co/C nanoboxes with good dispersion of Co nanoparticles. Hollow Co/C nanoboxes could promise good EM absorption performance, including minimum *RL* intensity of − 41.5 dB and broad EAB of 5.4 GHz even if its loading in paraffin matrix was very low (only 5 wt%). Such a performance was benefited from the synergy between Co nanoparticles and carbon component, as well as multiple reflections and scatters induced by hollow microstructure. In a similar study, Qiu et al. accomplished the encapsulation of Ni(OH)_2_/ZIF-67 with PDA layer and harvested hollow dodecahedral carbon capsules decorated with high-density CoNi alloy nanoparticles [168]. By adjusting the amount of Ni(OH)_2_, the chemical composition of CoNi/C composite could be easily tailored, and the optimized composite could consume EM waves in Ku band completely with the absorber thickness of 2.3 mm.

### Heterogeneous Contraction

In general, high-temperature pyrolysis may result in a contraction of organic frameworks and produce carbon-based counterparts with smaller particle size [169, 170]. However, if a heterogeneous coating is deposited on the surface of MOFs before high-temperature pyrolysis, the carbonization of organic frameworks will be different. For an inorganic coating, the carbonization of organic frameworks can be initiated at relatively low temperature and preferentially occur at those interfaces; for an organic polymer coating, its self-carbonization will be dominant in the initial stage [171, 172]. That is to say, no matter what kind of coating is applied, a new carbon layer will be in situ generated around MOFs crystals. This carbon layer not only plays an important role in stabilizing organic frameworks and resisting their contraction, but also provides nucleation sites for gaseous carbonaceous fragments released from interior MOFs cores. With the increase in pyrolysis degree, MOFs cores will be continuously damaged and finally disappear due to the accumulated stresses in their central parts [173], leading to the formation of hollow microstructure. Although both interfacial ion exchange and heterogeneous contraction can account for the cavitation in final carbon-based composites, their effect mechanisms are quite different, where interfacial ion exchange is more dependent on the chemical environment of MOFs crystals and heterogeneous contraction creates hollow microstructure during high-temperature pyrolysis. Silica and PDA are typical coatings that can work for heterogeneous contraction [173–176]. For example, our group previously conducted the polymerization of dopamine on the surface of FeCo PBAs [166], while interfacial ion exchange mentioned above was not observed because PBAs were more stable than ZIFs [166, 177]. The PDA coating was finally converted into hollow carbon nanocages that encapsulated core–shell FeCo@C nanoparticles derived from FeCo PBAs. EM measurements revealed that the optimal weight ratio of dopamine to FeCo PBAs was 0.75, and the minimum *RL* and the broadest EAB of the corresponding composite were − 67.8 dB and 5.3 GHz with the absorber thicknesses of 1.7 and 2.0 mm, respectively.

**Fig. 12:**
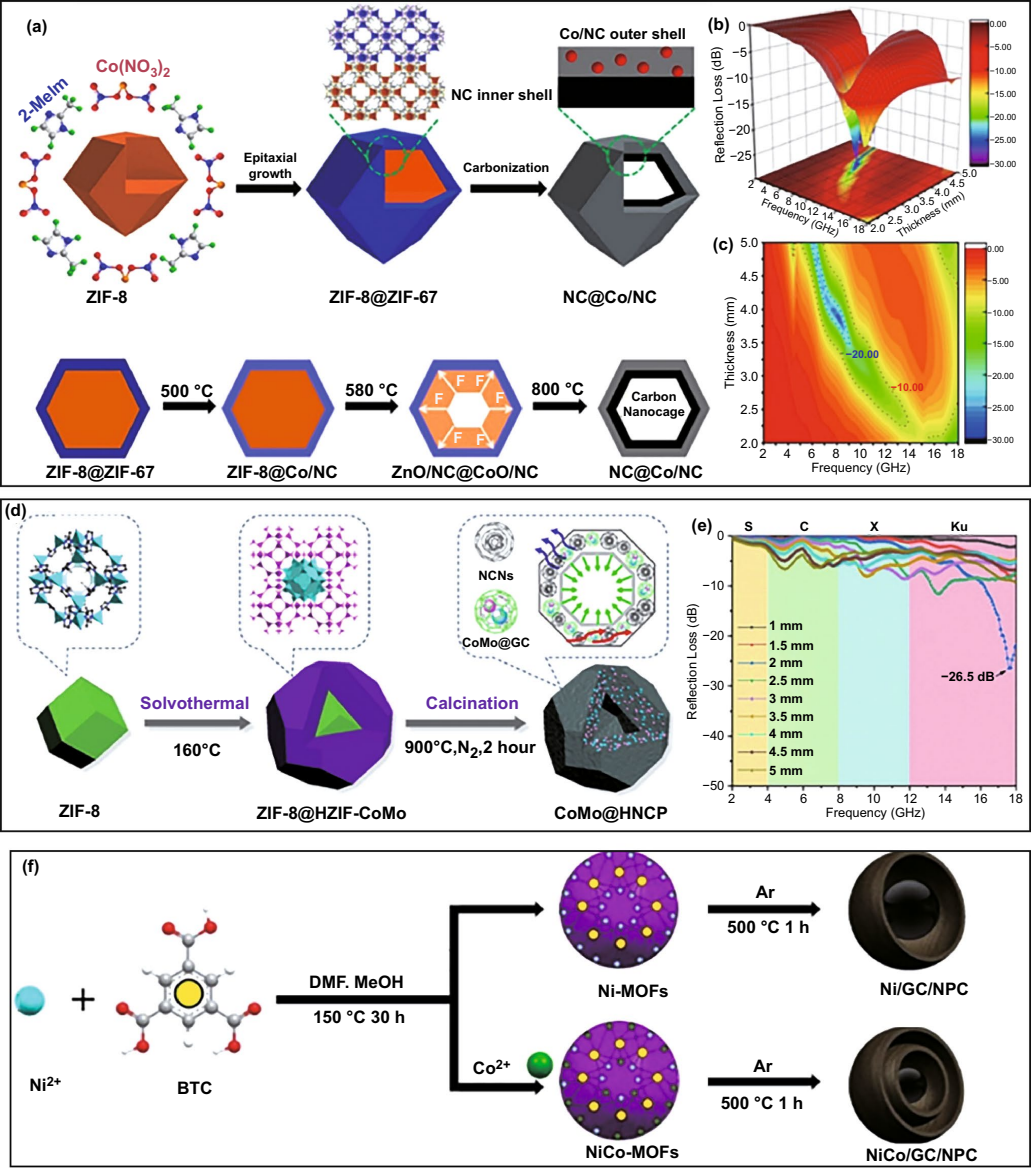
**a** Schematic illustration for the formation process and the synthetic mechanism of NC@Co/NC carbon nanocages*,* **b, c** 3D RL map and RL map of NC@Co/NC carbon nanocages. Reproduced with permission from Ref. [179]*.* Copyright *©* 2020 Elsevier B.V. **d** Schematic illustration for the synthesis process of hollow CoMo@HNCP polyhedron*,* **e** RL values of CoMo@HNCP. Reproduced with permission from Ref. [178]*.* Copyright *©* 2020 Elsevier Ltd. **f** Schematic formation process of the composites*.* Reproduced with permission from Ref. [182]*.* Copyright *©* 2020 Elsevier B.V.

Of note is that, in some cases, a heterogeneous MOFs layer can also display similar effect to silica or PDA [178]. For example, Liu’s group demonstrated the synthesis of hollow Co/N-doped carbon nanocages through thermal transformation of ZIF-8@ZIF-67 composites and investigated their EM properties (Fig. 12a) [179]. They found that ZIF-67 shell indeed counteracted the inward shrinkage of ZIF-8 polyhedrons during high-temperature pyrolysis and induced the formation of a hollow cavity in final composite. Hollow microstructure not only optimized impedance matching, but also enhanced conductive loss, intensified dipolar/interfacial polarization, and induced multi-scattering, resulting in the significant improvement in EM absorption performance as compared with solid N-doped carbon nanoparticles from direct pyrolysis of ZIF-8 polyhedrons. In particular, when the absorber thickness was 2.2 mm, the minimum *RL* intensity of hollow Co/N-doped carbon composite reached up to − 52.5 dB, and the corresponding EAB could cover the frequency range of 11.9–15.4 GHz (Fig. 12b, c). After the introduction of Mo species in outer ZIF-67 coating, they could further obtain hollow CoMo@N-doped carbon composite and broaden EAB in the whole Ku band with the absorber thickness of 2.0 mm (Fig. 12d, e) [178]. However, if ZIF-67 is chosen as the interior core instead, no hollow cavities can be observed in the pyrolysis products of ZIF-67@ZIF-8 polyhedrons [179, 180]. It is a consensus that ZIF-67 with relatively poor thermal stability may produce an inward adhesive force that impels the inward contraction of ZIF-8 shell with the increase in pyrolysis temperature [180]. Interestingly, Li et al. found a PDA layer on the surface of ZIF-67@ZIF-8 polyhedrons could reverse the situation and induce the formation of hollow microstructure in the resultant Co/C composites [181]. In addition, a few studies indicated that several specific MOFs could spontaneously change into magnetic carbon-based composites with hollow or yolk-shell microstructure during high-temperature pyrolysis, because the temperature gradient might cause the early carbonization of external surface and produce a carbon layer that could survive from the strain generated by inward contraction of organic frameworks [182]. For example, Xiong et al. directly fabricated yolk-shell Ni/C composites using low-crystalline and solid Ni-BTC microspheres as the precursor, and they even developed a double yolk-shell microstructure by the introduction of Co atoms in Ni-BTC (Fig. 12f) [182]. Double yolk-shell NiCo/C microspheres showed better EM absorption performance in both minimum *RL* intensity (− 52.2 dB vs. − 40.2 dB) and optimal EAB (7.2 GHz vs. 3.4 GHz) with the absorber thickness of 2.1 mm, demonstrating the positive effects on composition optimization and microstructure design. It is unfortunate that this simple strategy only works in some random reports, and the corresponding universality still needs to be explored.

## Overview about EM Absorption Performance of MOFs-derived Magnetic Carbon-Based Composites

Most of the above examples demonstrate that rational manipulation on the composition and microstructure of MOFs-derived magnetic carbon-based composites can make a solid contribution to their EM absorption enhancement. As we all know, an excellent microwave absorber should have four key features, namely strong absorption, wide frequency response, thin applied thickness, and lightweight (low filler loading). In Table 1, we list these parameters of some representative candidates from the categories mentioned above to intuitively predict their prospects as high-performance microwave absorbers. One can easily find that almost all microwave absorbers in this table can produce very strong RL intensity less than − 60.0 dB, suggesting either composition optimization or microstructure design will be highly effective for deep conversion of EM energy. Meanwhile, some of them, e.g., FeCoNi@C microsphere [68], FeCo/NC/rGO composite [85], FeCo/C@WC aerogel [154], yolk-shell Co–C/Void/Co_9_S_8_ [163], and yolk-shell NiCo/GC/NPC [182], even promise considerable EAB over 7.0 GHz, which further highlights the universality of these two kinds of modification strategies in widening absorption frequency. However, such excellent EM absorption performance of FeCoNi@C microsphere and FeCo/NC/rGO composite is achieved with a relatively high filler loading or a relatively large applied thickness [68, 85], and in contrast, FeCo/C@WC aerogel shows fantastic RL intensity and EAB with the filler loading and applied thickness of 15 wt% and 1.9 mm, respectively. Based on four features mentioned above, FeCo/C@WC aerogel is one of MOFs-derived candidates with the best practical application prospects. The significant reinforcement in EM absorption can be explained from the following aspects: (1) the proper weight ratio of magnetic nanoparticles to carbon components can efficiently regulate the gap between relative complex permittivity and complex permeability, which improves the impedance matching effectively; (2) the involvement of aerogel microstructure is not only conducive to the formation of conductive network, promoting the migration and hopping of electrons and consolidating conductivity loss, and meanwhile, it also brings high dispersion of magnetic nanoparticles, reducing the possibility of skin effect and developing full capability of magnetic components; (3) the extremely high porosity in aerogel increases the propagation distance of incident EM waves and induces multiple reflections of incident EM waves, so that the final composite can realize good performance with both low filler loading and small applied thickness; (4) the abundant heterogeneous interfaces and various defects in carbon frameworks are greatly favorable for the accumulation of various polarization, especially for interfacial polarization and dipole orientation polarization, supplying more consumption paths for incident EM waves.

**Table 1:** EM absorption performance of some representative MOFs-derived magnetic carbon-based composites

MOF precursors	Modificated strategies	Samples	*RL* values (dB) [frequency (GHz), thickness (mm)]	EAB (GHz) [range (GHz), thickness (mm)]	Filler loading (wt%)	Refs.
Co-MOFs	Glucose additive	Co/C micropheres	− 71.3 (6.2, 3.8)	6.6 (11.3–17.9, 2.0)	60	[9]
CoNi-BTC	rGO additive	CoNi@NC/rGO composite	− 68.0 (10.9, 3.0)	6.7 (11.3–18.0, 2.5)	25	[83]
MOF-74	Magnetic additive	FeCoNi@C microsphere	− 64.8 (15.4, 2.1)	8.1 (9.9–18.0, 2.5)	38	[68]
Fe-doped Co-MOFs	rGO additive	FeCo/NC/rGO composite	− 43.3 (11.3, 2.5)	9.3 (8.7–18.0, 2.6)	25	[85]
ZIF-67	MF sponge template	Co/CNTs/CS sponge	− 51.2 (12.0, 2.2)	4.1 (10.3–14.4, 2.2)	5	[149]
ZIF-67	Melamine foam template	MZ800 foam	− 59.8 (12.9, 2.3)	5.6 (12.4–18.0, 2.1)	20	[147]
ZIF-67	Wood aerogel template	FeCo/C@WC aerogel	− 47.6 (15.7, 1.5)	8.9 (9.1–18.0, 1.9)	15	[154]
FeCo PBAs	PDA coating	Hollow FeCo@C cage	− 67.8 (5.3, 1.7)	5.3 (11.0–16.3, 2.0)	50	[166]
NiCo PBAs	Chemical etching	Hollow NiCo@C microbox	− 68.4 (13.4, 2.1)	5.8 (12.2–18.0, 2.0)	40	[134]
ZIF-67	CoNi-LDH coating	Hollow CoNi/C composite	− 61.0 (13.7, 2.0)	5.2 (12.8–18.0, 2.0)	10	[157]
ZIF-67	Chemical etching	Hollow Co/C cage	− 60.6 (9.8, 2.4)	5.1 (10.8–15.9, 1.9)	10	[129]
ZIF-67	PDA coating	Hollow Co/N/C@MnO_2_	− 58.9 (12.0, 3.7)	5.5 (9.5–15.0, 1.9)	15	[100]
ZIF-67	Cobalt sulfide coating	Yolk-shell Co–C/Void/Co_9_S_8_	− 54.0 (3.0, 4.9)	8.2 (9.8–18.0, 2.2)	25	[163]
NiCo-MOFs	Gradient carbonization	Yolk-shell NiCo/GC/NPC	− 52.2 (7.2, 2.1)	7.2 (10.8–18.0, 2.1)	30	[182]

In order to further address the advantage of MOFs-derived strategy, EM absorption performance of some typical microwave absorbers prepared from different methods is also listed in Table 2, where rGO- and MXene-based composites, two kinds of the most attractive candidates, are taken as the primary references. Benefiting from unique two-dimensional structure, individual rGO or MXene can produce EM absorption ability to some extent, and especially for Ti_3_C_2_T_*x*_, its minimum RL intensity and EAB are − 40.0 dB and 6.8 GHz, respectively, with the absorber thickness of 2.0 mm and the filler loading of 50 wt% [183, 184]. However, high-temperature treatment usually causes remarkable degradation in EM absorption performance of Ti_3_C_2_T_*x*_, which may be attributed to the stacking of two-dimensional nanosheets [185]. The introduction of magnetic nanoparticles cannot only suppress the stacking of rGO or MXene nanosheets, but also supplement salutary magnetic loss mechanism in final composites. As a result, both magnetic rGO-based composites and magnetic MXene-based composites displayed more or less EM enhancement [186–189]. It is worth noting that CoNi/rGO and FeCo/Ti_3_C_2_T_*x*_ even generate very broad EABs of 7.3 and 8.8 GHz with the absorber thickness of 2.0 and 1.6 mm, respectively, while these achievements require high filler loading over 60 wt% [190, 191]. In contrast, some microwave absorbers from MOFs-derived strategy, such as FeCo/C@WC aerogel, CoNi@NC/rGO, and FeCo/NC/rGO, exhibit equal or even broader EABs, and meanwhile, their RL intensities are also smaller than those of conventional composites [83, 85, 154]. More importantly, their filler loadings are obviously decreased to no more than 30 wt%, demonstrating the superiority of MOFs-derived strategy intuitively. The significant EM reinforcement can be attributed to MOFs characteristics as we mentioned above: on the one hand, their periodic atom arrangements promote uniform dispersion of magnetic nanoparticles, which fully develops their intrinsic magnetic loss, and on the other hand, the good chemical homogeneity of MOFs-derived carbon composites always induces various polarization relaxations favorable for EM attenuation. In addition, the introduction of various additives, such as rGO and wood aerogel, also establishes some microstructure advantage to intensify energy conversion of incident EM waves.

**Table 2:** Comparison of EM absorption performance for some representative microwave absorbers from various preparation methods

Samples	Preparation	*RL* values (dB) [frequency (GHz), thickness (mm)]	EAB (GHz) [range (GHz), thickness (mm)]	Filler loading (wt%)	Refs.
rGO sheet	Chemical reduction method	− 37.2 ( 5.9, 3.5)	2.5 (8.0–10.5, 3.5)	30	[183]
Ti_3_C_2_T_x_	Mixture pyrolysis	− 40.0 (7.8, 2.0)	6.8 (11.2–18.0, 2.0)	50	[184]
Annealed-Ti_3_C_2_T_x_	Calcination	− 48.4 (11.6, 1.7)	2.8 (9.5–12.3, 1.9)	50	[185]
Ni/graphene composite	Atomic layer deposition method	− 22.1 (15.0, 2.0)	4.0 (12.1–16.1, 2.0)	10	[186]
Fe/graphene composite	Hydrothermal method	− 45.0 (7.1, 3.0)	4.4 (9.9–14.3, 2.0)	40	[187]
Ni-modified Ti_3_C_2_T_x_	Decoration	− 24.9 (11.2, 2.0)	6.3 (11.7–18.0, 1.5)	Unknown	[188]
Ti_3_C_2_T_x_/Ni chain	Decoration	− 49.9 (11.9, 1.8)	2.1 (10.9–13.0, 1.8)	50	[189]
CoNi/rGO composite	Decoration	− 31.0 (4.9, 4.0)	7.3 (9.5–16.8, 2.0)	60	[190]
Ti_3_C_2_T_x_/FeCo composite	Decoration	− 17.9 (9.3, 1.6)	8.8 (9.2–18.0, 1.6)	70	[191]
CoNi@NC/rGO composite	MOFs-derived method	− 68.0 (10.9, 3.0)	6.7 (11.3–18.0, 2.5)	25	[83]
FeCo/NC/rGO composite	MOFs-derived method	− 43.3 (11.3, 2.5)	9.3 (8.7–18.0, 2.6)	25	[85]
FeCo/C@WC aerogel	MOFs-derived method	− 47.6 (15.7, 1.5)	8.9 (9.1–18.0, 1.9)	15	[154]

## Conclusion and Outlooks

Herein, the recent developments of various strategies on elaborate composition and microstructure design in MOFs-derived magnetic carbon-based composites, together with their promising applications in EM absorption, are summarized in detail. It is undoubted that composition optimization is indeed favorable for the reinforcement of microwave absorption performance by improving impedance matching and EM characteristics of final composites, and microstructure upgradation brings many additional effects, including the formation of conductive networks and the substantial extension in propagation distance of incident EM waves, as well as more powerful dipole orientation polarization and interfacial polarization.

Although some breakthroughs have been witnessed in the synthesis and application for EM absorption of MOFs-derived magnetic carbon-based composites with tunable chemical compositions and various microstructures, this research field still remains many challenges. First, the combination of carbon components and magnetic nanoparticles can overcome the shortcomings of individual counterparts and produce a synergistic effect to upgrade EM absorption performance, while most composites with optimized ratio of magnetic and carbon components are only active in the frequency range of 8.0–18.0 GHz. This situation seriously hinders their practical application in the field of electronics industry since the effective working frequency of many electronic devices is usually lower than 8.0 GHz, and thus, the composition optimization for low-frequency absorption is urgently developed. Second, the state of the art in microstructure design is usually dependent on some assisted strategies (e.g., etching, templates, and SiO_2_/polymer coating) and involves complex multi-step processes, which set an obstacle for their practical application. A simple strategy is still desirable for microstructure upgradation in MOFs-derived magnetic carbon-based composites. For example, sonochemistry has displayed its advantages in the creation of various unique microstructures in MOFs derivatives by breaking the dimensional limitation and controlling the thickness of shell, while it is still inaccessible in the field of EM absorption. Third, it is well known that the frameworks of MOFs crystals are designable and can be incorporated with different metal ions/clusters and organic linkers during the self-assembly process, and thus, it will be expected to obtain high-performance microwave absorbers from various MOFs. However, the current works mainly concentrate on ZIFs, PB or MIL series derivatives, which means other novel MOFs may reveal new outcomes to enrich the diversity of microwave absorbers. Fourth, there are hundreds of papers on EM absorption of magnetic carbon-based composites with different microstructures published in recent years, and most of them lack the in-depth understanding about the microstructure-property relationship. A comprehensive investigation on attenuation mechanism of different microstructure to EM waves will be greatly helpful for readers to understand how to design the microstructure of their samples. Fifth, performance is just one of the requirements in practical application, and besides, environmental tolerance is another important character for microwave absorbers to keep their durability. The encapsulation of magnetic nanoparticles on carbon matrix may be efficient to improve the environmental tolerance of magnetic carbon-based composites. What’s more, in terms of current market prospect, high cost of MOFs is an inevitable barrier for the commercialization of their derivatives, and thus, the search of an effective strategy for low-cost mass production is also a challenging and in high demand task. When these problems are solved one by one with the tireless efforts of global researchers, novel magnetic carbon-based composites with reasonable compositions and elaborate microstructures from MOFs will exhibit a bright prospect as high-performance microwave absorbers for EM pollution precaution.
